# External validation of a rapid, non-invasive tool for periodontitis screening in a medical care setting

**DOI:** 10.1007/s00784-021-03952-2

**Published:** 2021-05-12

**Authors:** N. Nijland, F. Overtoom, V. E. A. Gerdes, M. J. L. Verhulst, N. Su, B. G. Loos

**Affiliations:** 1 Present Address: Department of Periodontology, Academic Center for Dentistry Amsterdam (ACTA), University of Amsterdam and Vrije Universiteit Amsterdam, Gustav Mahlerlaan 3004, 1081 LA Amsterdam, The Netherlands; 2 Department of Vascular Medicine, Amsterdam University Medical Center, Amsterdam, The Netherlands; 3 Sunstar Suisse, Etoy, Switzerland; 4 Department of Oral Public Health, Academic Center for Dentistry Amsterdam (ACTA), University of Amsterdam and Vrije Universiteit, Amsterdam, The Netherlands

**Keywords:** Periodontitis, Self-reported oral health, Questionnaire, Screening, Medical care, Validation

## Abstract

**Objectives:**

Medical professionals should advise their patients to visit a dentist if necessary. Due to the lack of time and knowledge, screening for periodontitis is often not done. To alleviate this problem, a screening model for total (own teeth/gum health, gum treatment, loose teeth, mouthwash use, and age)/severe periodontitis (gum treatment, loose teeth, tooth appearance, mouthwash use, age, and sex) in a medical care setting was developed in the Academic Center of Dentistry Amsterdam (ACTA) [1]. The purpose of the present study was to externally validate this tool in an outpatient medical setting.

**Materials and methods:**

Patients were requited in an outpatient medical setting as the validation cohort. The self-reported oral health questionnaire was conducted, demographic data were collected, and periodontal examination was performed. Algorithm discrimination was expressed as the area under the receiver operating characteristic curve (AUROCC). Sensitivity, specificity, and positive and negative predictive values were calculated. Calibration plots were made.

**Results:**

For predicting total periodontitis, the AUROCC was 0.59 with a sensitivity of 49% and specificity of 68%. The PPV was 57% and the NPV scored 55%. For predicting severe periodontitis, the AUROCC was 0.72 with a sensitivity of 54% and specificity of 81%. The PPV was 34% and the NPV 81%.

**Conclusions:**

The performance of the algorithm for severe periodontitis is found to be sufficient in the current medical study population. Further external validation of periodontitis algorithms in non-dental school populations is recommended.

**Clinical relevance:**

Because general physicians are obligated to screen patients for periodontitis, it is our general goal that they can use a prediction model in medical settings without an oral examination.

**Supplementary Information:**

The online version contains supplementary material available at 10.1007/s00784-021-03952-2.

## Introduction

Periodontal disease is a common non-communicable disease. Approximately 30–50% of adults suffer from some form (mild, moderate, or severe) of periodontitis. The prevalence of severe periodontitis is estimated at 9–11% [2–5]. Notably, it has been established that periodontitis has an evident bi-directional link with diabetes mellitus [6, 7] and this fact underlines the need for dental professionals as well as medical professionals to know whether their patients may be suffering from one or the other condition or both.

Diabetes mellitus (type 1 and type 2 combined) is a globally spread disease of which around 451 million people suffered from in 2017 (age 18–99 years). The worldwide prevalence of diabetes mellitus is expected to rise to 693 million patients by 2045. Diabetes mellitus increases the susceptibility and severity of periodontal diseases [7–9]. Studies have reported the higher prevalence of periodontitis in diabetes mellitus patients compared to healthy individuals [1, 10]. On the other hand, severe periodontitis patients show a significantly higher prevalence of diabetes mellitus (12.8%) compared to patients without periodontitis (5.45%) [11]. It is more challenging for diabetes mellitus patients with periodontitis to maintain stable glucose plasma levels [12]. As such, periodontal health improvement can improve metabolic control of diabetes mellitus patients. For example, whole mouth subgingival scaling and additional periodontal surgery showed to reduce the plasma HbA1c levels significantly in patients with type 2 diabetes mellitus ánd having moderate to severe periodontitis [13]. Thus, treatment of periodontitis and routine oral health assessment may be essential for effective management of type 2 diabetes mellitus [13–15].

The bi-directional link between diabetes mellitus and periodontitis has motivated the International Diabetes Federation (IDF) to develop oral health guidelines for diabetes care professionals [16]. These recommendations were followed in the Netherlands by the association of general physicians with the following national diabetes mellitus care guideline: “The family physician inspects the mouth and pays attention to signs of periodontitis. He advises the patient to visit the dentist/ oral hygienist twice a year” [17]. Medical professionals should advise and stimulate their diabetes mellitus patients to visit a dentist if necessary. Nonetheless, due to the lack of time and knowledge, a good inspection of the oral cavity is not performed and screening for periodontitis is difficult in the medical setting. To alleviate this problem, a rapid and non-invasive tool for periodontitis screening in a medical care setting was developed [1]. By using this tool, medical professionals can, without performing any oral inspection, simply inform their patients whether they are suspected of having periodontitis and advise them to visit a dentist for further diagnostic procedures.

The rapid and non-invasive screening tool for periodontitis based on a few patient demographic data and answers to 8 Self-Reported Oral Health (SROH) questions [18] was developed in the Academic Center of Dentistry Amsterdam (ACTA), and included a dental school population [1]; a full-mouth clinical periodontal examination was used as gold standard. The authors classified periodontitis as moderate or severe with the CDC-AAP classification. The prediction algorithm based on age and a few questions on the perception of oral health proved sufficiently accurate to suspect moderate and severe periodontitis. This suggested that the screening tool is easily applicable and ideally intended for the non-dental setting. However, the tool was developed in a dental school and external validation of the tool is not yet available. Therefore, the purpose of the present study was to externally validate the rapid non-invasive tool for the screening of periodontitis in a medical setting.

## Methods

### Study size

Because this study was set up as a pilot, no a priori power analysis was carried out.

### Compliance with ethical standards

The present study is carried out as a cross-sectional research. The external validation of two algorithms (for moderate and severe periodontitis) from Verhulst et al. is done in a medical care setting to find out if they can perform well outside of the ACTA dental clinic [1].

The study was performed in an outpatient medical setting by recruiting patients of the internal medicine policlinic in the Amsterdam UMC (University Medical Center), location AMC (Academic Medical Center) as the validation cohort. Within 1 week before the clinical visit at the policlinic, all planned patients received an information letter containing research information. After arrival at the internal medicine policlinic, patients were asked if they were willing to participate; if so, informed consent was signed. Everybody was screened once. Patients ≥18 and ≤ 80 years of age and with at least one of their own teeth were suitable. Patients <18 and > 80 years of age and edentulous patients with or without full dentures (regardless of dental implant support) were excluded.

First, the self-reported oral health (SROH) questionnaire was conducted in the same way as the previous study [1]. The questionnaires were coded beforehand with research numbers to insure anonymity. Second, demographic data were collected. Finally, the periodontal examination was performed.

A key document connected the research numbers to the AMC patient numbers. This document was stored together with the completed questionnaires in separate folders in a closed closet at the Department of Periodontology at ACTA.

### Self-reported oral health questionnaire

The SROH questionnaire consists of 8 questions totally (Supplementary Table 1). All questions are closed-ended and were made dichotomous. Five questions (Q1 and Q3–Q6) are dichotomous with “yes” or “no” answer possibilities. Q2 asks the patient to rate his/her dental health on a 5-point scale and was made dichotomous (combined reference category [18]) with a negative (poor and fair) or positive (good, very good, and excellent) answer. Q7 asks how often the patient uses interdental products. Q8 asks the same but for mouthwash/oral rinse products. Both Q7 and Q8 are expressed as number of days per week. Q7 (floss use: 1–7 days per week or never) and Q8 (mouthwash use: 1–7 days per week or never) were made dichotomous as well [1].

### Periodontal examination

Since a full-mouth clinical periodontal examination as gold standard in the hospital outpatient clinic was not feasible, we resorted to the use of intra-oral screening of the periodontal condition applying the Community Periodontal Index of Treatment Needs (CPITN) as alternative gold standard [19]. The CPITN was performed by authors NN and FO. Per sextant, the deepest measured pocket and bleeding on probing are used to score the CPITN, being either 0–2, 3, or 4. In this study, the group with any score CPITN score 3 and/or 4 combined was seen as total periodontitis and the group with CPITN score 4 was seen as severe periodontitis. The periodontal examiner was blinded for the outcomes of SROH questionnaire and demographic patient data. Patients were informed about their periodontal state of health. When the CPITN score was 4, an information letter was given, and the patient was encouraged to visit the dentist.

### Statistical analysis

The statistical analysis was performed using IBM SPSS statistics, v26 (IBM, New York, NY, USA). The descriptive background data were analyzed by one-way ANOVA or a chi-square test to find differences in variables between the CPITN groups. A chi-square test was also used to analyze categorical data from the SROH questions and odds ratios (OR) and confidence intervals (CI) were calculated. The *p*-values for all variables were calculated and *p*-value <0.05 was set as statistically significant.

In order to use the prediction model, all items were dichotomized as described above. A reference outcome was set a priori and coded with either 0 (a negative outcome) or 1 (a positive outcome). The age data were dichotomized in <40 years of age and ≥ 40 years of age as in the study from Verhulst et al. [1].

By using the multivariate binary logistic regression model with backward selection by likelihood ratio method, Verhulst et al. [1] set up algorithms with the following formula: *Y* = *B*_1_*X*_1_ +... + *B*_*n*_*X*_*n*_. *Y* is the individual sum score, and *B* is the regression coefficient of a predictor in the model. The *X* for a negative category of a predictor was coded as 0 and a positive category of a predictor as 1. The algorithm for total periodontitis was: *Y* = 1.692*Q2 + 1.286*Q3 + 1.560*Q4 + 1.075*Q8 + 2.209*Age. For severe periodontitis, it was *Y* = 2.073*Q3 + 1.277*Q4 + 1.590*Q6 + 1.440*Q8 + 1.615*Age + 1.091*Sex [1].

For every individual, the sum score of the algorithm was calculated by filling in the predictors. After that, the individual predicted probability was calculated by 1−1/[1+exp(constant+ *B*_1_*X*_1_ + … + *B*_*n*_*X*_*n*_)] and saved as a new variable. This procedure was performed for both algorithms. The constant for total periodontits was − 2.368 and for severe periodontitis − 4.763. The constants are calculated by performing the logistic regression analysis with backward selection by likelihood ratio as described above.

The discrimination of the algorithms was expressed as the area under the receiver operating characteristic curve (AUROCC) and displayed the ability of the algorithm to distinguish “suspected to have (severe) periodontitis” versus “not suspected to have (severe) periodontitis.” The graph was derived by plotting the individual predicted probability against the measured dichotomized CPITN score. Because the characteristics of the current study population and previous study population may be different (in demographic characteristics and health status), the optimal predicted probability was calculated again. The optimal predicted probability cut-off value was identified by defining the highest sum of the sensitivity and specificity that could be found on the ROC curve. Calibration and discrimination are the most important parameters we used to assess the external validity of the model. The calibration and discrimination do not change when the cut-off values are different. With the new cut-off value, the misclassification of the patients based on the prediction model is the smallest in the current study population. Calibration of the algorithms was assessed by plotting the predicted individual probability against the observed actual risk in calibration plots.

When the individual predicted probability was higher than the optimal predicted probability cut-off value, the patient was likely to have (severe) periodontitis. When the individual predicted probability was lower than the optimal predicted probability cut-off value, the patient was likely not to have (severe) periodontitis. Herewith, a new binary value which represented the predicted periodontal state was made. Sensitivity, specificity, and positive and negative predictive values (PPV and NPV) were calculated.

## Results

### Patients’ characteristics

One hundred fifty-nine patients were recruited in this study. Of these, 1 was edentulous and 3 appeared not to be a patient of the internal medicine policlinic and for this reason excluded from the study, leaving 155 patients included (Table 1.). From these, 69 (44.5%) patients had CPITN score 0–2, 62 (40%) CPITN score 3 and 24 (15.5%) CPITN score 4. The mean age of the population was 55.7 ± 15.6 years. Eighty-five (54.8%) males and 70 (45.2%) females participated. Twenty-one (13.5%) patients were current smoker and 29 (18.7%) had diabetes mellitus. The proportion of the patients ≥40 years of age is significantly higher in CPITN score 4 group than in the other CPITN groups (*p* = 0.045). The mean age of the patients with CPITN score 0–2 was 52.9 ± 17.5 years of age, with CPITN score 3 56.4 ± 14.6 years of age, while patients in CPITN score 4 were on average older with 61.6 ± 9.4 years of age. However, the mean age was not significantly different between the groups (*p* = 0.057). Patients with CPITN score 4 were more likely to be a smoker (*p* = 0.009) compared to patients with CPITN score 0–2 and 3. The male/female distribution and the frequency diabetes mellitus did not show any statistically significant differences between the CPITN groups.

**Table 1.** Description of the study population divided by CPITN scores

	Total study population	CPITN 0-2	**CPITN 3**	CPITN 4	*p*-value^a^
*N* (%)	155 (100)	69 (44.5)	62 (40)	24 (15.5)	
Age (years)	55.7 ± 15.6	52.9 ± 17.5	56.4 ± 14.6	61.6 ± 9.4	0.057
Age dichotomized					
<40 years	25 (16.1)	15 (21.7)	10 (16,1)	0 (0)	0.045^*^
≥40 years	130 (83.9)	54 (78.3)	52 (83.9)	24 (100)	
Sex					
Male	85 (54.8)	33 (47.8)	39 (62.9)	13 (54.2)	0.223
Female	70 (45.2)	36 (52.2)	23 (37,1)	11 (45.8)	
Smoking (current)	21 (13.5)	7 (10.1)	6 (9.7)	8 (33.3)	0.009^*^
Diabetes mellitus	29 (18.7)	9 (13)	13 (21)	7 (29.2)	0.116

Data are presented as either mean ± SD or *n* (%)

^a^Differences between the three CPITN groups were tested by one-way ANOVA (continuous data) or chi-square test (categorical data)

^*^Statistically significant with *p* < 0.05

### Self-reported oral health

The responses to three questions, namely “Do you think you might have gum disease” (Q1) (*p* = 0.001; OR 3.751; 95% CI: 1.669–8.432), “Overall, how would you rate the health of your teeth and gums?” (Q2) (p = 0.003; OR 3.66; 95% CI: 1.49–8.96), “Have you ever had treatment for gum disease such as scaling and root planing, sometimes called ‘deep cleaning’?” (Q3) (*p* = <0.001; OR 4.70; 95% CI: 1.88–11.76), and “Have you ever had any teeth become loose on their own, without an injury?” (Q4) (p = 0.044; OR 2.76; 95% CI: 1.00–7.64) were significantly associated with CPITN score 4 (Table 2.). The remaining questions (Q1 and Q5–Q8) did not show any significant difference across the different CPITN groups.

**Table 2.** Responses to the self-reported oral health (SROH) questionnaire

SROH item	Response *n* (%)	CPITN 0-3 *n* (%)	CPITN 4 *n* (%)	*p*-value^a^	OR (95% CI)^b^
Q1. *Do you think you might have gum disease?*					
Yes^†^	35 (22.6)	27 (20.6)	8 (33.3)	0.171	1.93 (0.75–4.97)
No	120 (77.4)	104 (79.4)	16 (66.7)		
Q2. *Overall, how would you rate the health of your teeth and gums?*					
Poor, fair^†c^	45 (29)	32 (24.4)	13 (54.2)	0.003^*^	3.66 (1.49–8.96)
Good, very	110 (71)	99 (75.6)	11 (45.8)		
good,					
excellent^†c^					
Q3. *Have you ever had treatment for gum disease such as scaling and root planing, sometimes called “deep cleaning”?*					
Yes^†^	35 (22.6)	23 (17.6)	12 (50)	<0.001^*^	4.70 (1.88–11.76)
No	120 (77.4)	108 (82.4)	20 (50)		
Q4. *Have you ever had any teeth become loose on their own, without an injury?*					
Yes^†^	24 (15.5)	17 (13)	7 (29.2)	0.044^*^	2.76 (1.00–7.64)
No	131 (84.5)	114 (87)	17 (70.8)		
Q5. *Have you ever been told by a dental professional that you lost bone around your teeth?*					
Yes^†^	36 (23.2)	28 (21.4)	8 (33.3)	0.202	1.84 (0.71–4.74)
No	119 (76.8)	103 (78.6)	16 (66.7)		
Q6. *During the past three months, have you ever noticed a tooth that doesn’t look right?*					
Yes^†^	22 (14.2)	18 (13.7)	4 (16.7)	0.706	1.26 (0.39–4.10)
No	133 (85.8)	113 (86.3)	20 (83.3)		
Q7. *Aside from brushing your teeth with a toothbrush, in the last seven days, how many times did you use dental floss or any other device to clean between your teeth?*					
1–7 days/wk.^†^	132 (85.2)	113 (86.3)	19 (79.2)	0.369	0.61 (0.20–1.83)
Never	23 (14.8)	18 (13.7)	5 (20.8)		
Q8. *Aside from brushing your teeth with a toothbrush, in the last seven days, how many times did you use mouthwash or other dental rinse product that you use to treat dental disease or dental problems?*					
1–7 days/wk.^†^	46 (29.7)	37 (28.2)	9 (37.5)	0.362	1.52 (0.61–3.79)
Never	109 (70.3)	94 (71.8)	15 (62.5)		

^a^Differences between the three CPITN groups were analyzed by using chi-Square tests

^b^Odds ratios (OR) and confidence intervals (CI) were calculated with reference categories as indicated (^†^)

^c^Combined reference category, according to Eke et al. [18]

^*^Statistically significant with *p* < 0.05

### Validation of the algorithm

**Fig. 1:**
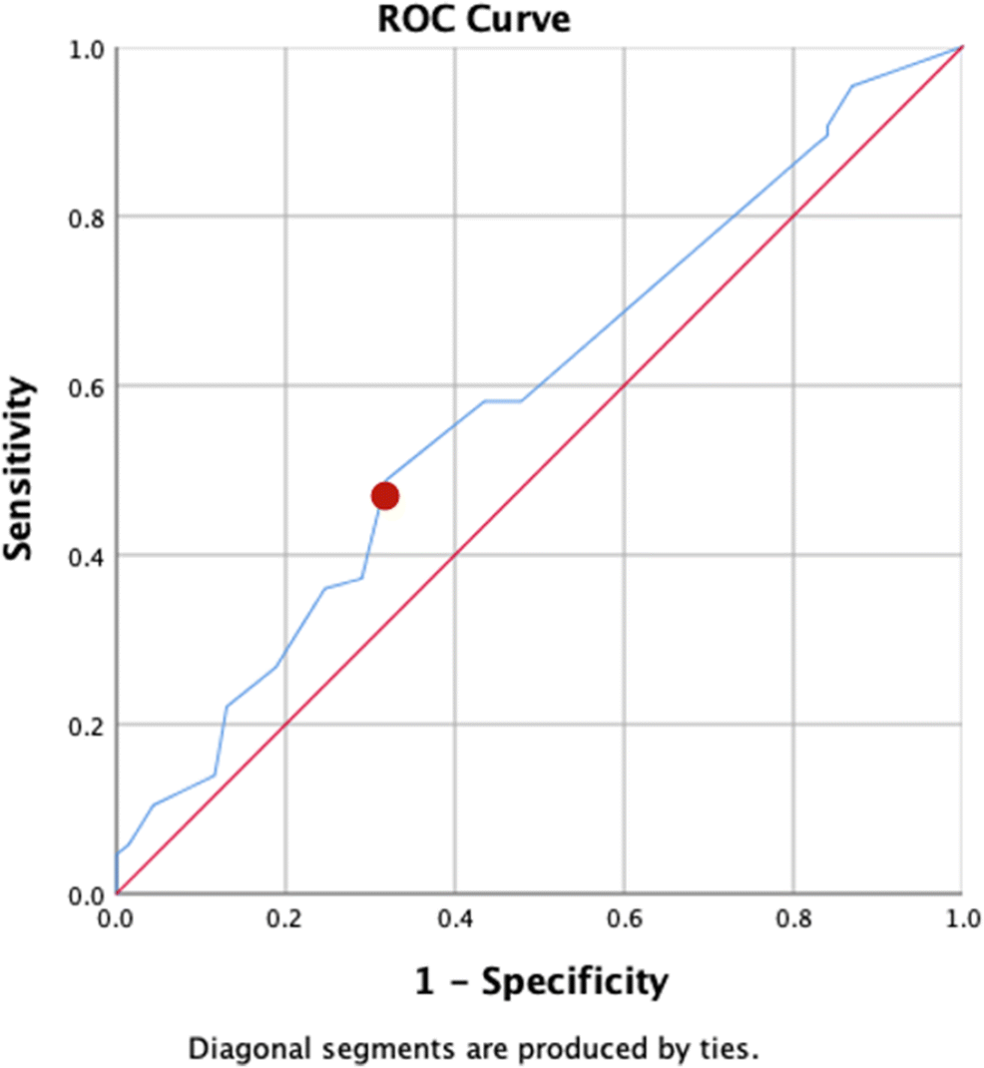
ROC curve of the algorithm (*Y* = 1.692*Q2 + 1.286*Q3 + 1.560*Q4 + 1.075*Q8 + 2.209*Age) from Verhulst et al. [1] to predict total periodontitis with AUROCC 0.59 (95% CI: 0.50–0.68). The dot indicates the predicted probability cut-off value of 0.34 with sensitivity of 49% and specificity of 68%

**Fig. 2:**
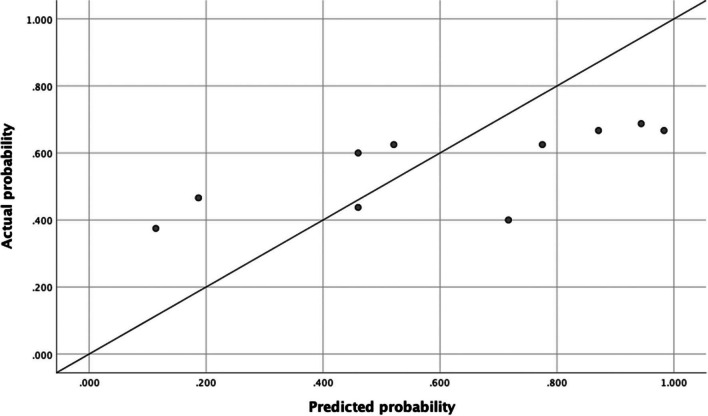
Calibration plot of the algorithm for the total periodontitis. The algorithm from Verhulst et al. [1] *for total periodontitis is Y* = 1.692*Q2 + 1.286*Q3 + 1.560*Q4 + 1.075*Q8 + 2.209*Age. The reference line is what would result if the predicted probability was the same as the actual probability of the model so that the prediction is neither underestimated nor overestimated

Table 3 presents the algorithm performances. By applying the model from Verhulst et al. [1] for prediction of total periodontitis in the current study population, the following results were found. The ROC curve is displayed in Fig. 1. For predicting CPITN score 3 and 4 combined (total periodontitis), the AUROCC was 0.59 (95% CI 0.50–0.68; SD 0.05). The optimal predicted probability cut-off value was 0.34. The calibration plot showed that most of the dots were not lying close to the reference line, which indicated that there was not a good fit between the predicted probability and the actual probability (Fig. 2). The sensitivity of the algorithm in this population at the optimal score was 49% and the specificity was 68%. The PPV was 57% and the NPV scored 55%.

**Table 3:** Performances of algorithms for total and severe periodontitis in the current study population

Algorithm performances^a^		
	Total periodontitis (CPITN score 3 and 4)	Severe periodontitis (CPITN score 4)
AUROCC (95% CI)	0.59 (0.50–0.68)	0.72 (0.61–0.83)
Optimal predicted probability	0.34	0.41
Sensitivity (%)	49	54
Specificity (%)	68	81
PPV (%)	57	34
NPV (%)	55	81

^a^Developed by Verhulst et al. [1]

**Fig. 3:**
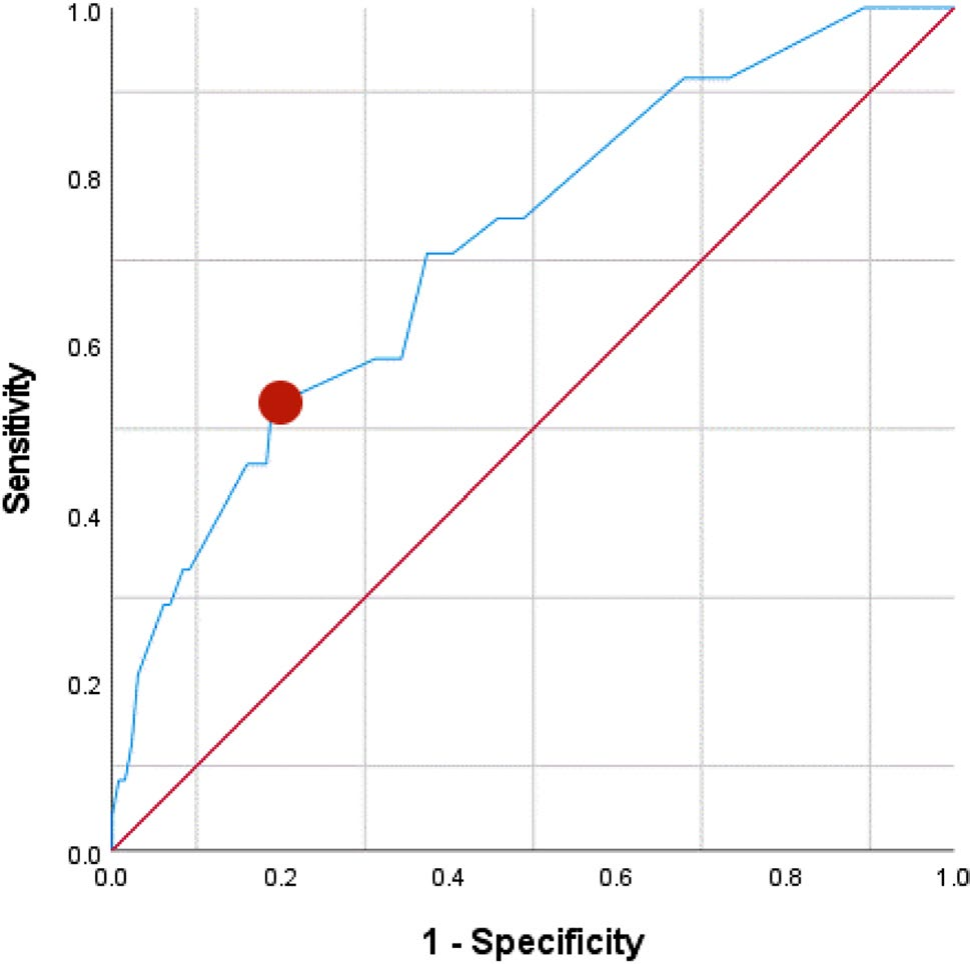
ROC curve of the algorithm from Verhulst et al. [1] *to predict severe periodontitis (Y* = 2.073*Q3 + 1.277*Q4 + 1.590*Q6 + 1.440*Q8 + 1.615*Age + 1.091*Sex) with AUROCC 0.72 (95% CI: 0.61–0.823). The dot indicates the optimal predicted probability cutoff value of 0.41 with sensitivity of 54% and specificity of 81%

**Fig. 4:**
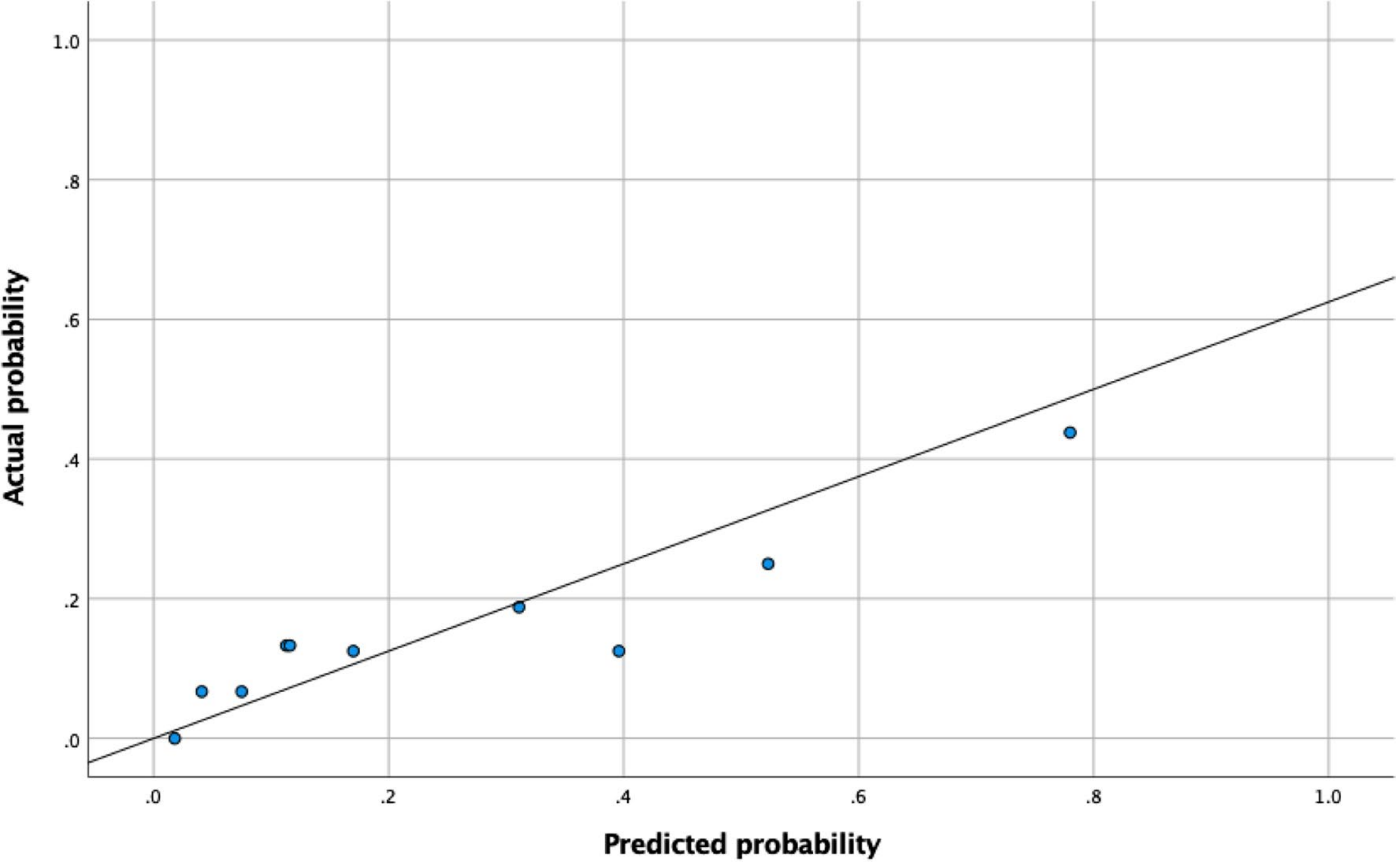
Calibration plot of the algorithm for severe periodontitis. The algorithm from Verhulst et al. [1] *for severe periodontitis is Y* = 2.073*Q3 + 1.277*Q4 + 1.590*Q6 + 1.440*Q8 + 1.615*Age + 1.091*Sex. The reference line is what would result if the predicted probability was the same as the actual probability of the model so that the prediction is neither underestimated nor overestimated

The ROC curve for the severe periodontitis algorithm is displayed in Fig. 3. For predicting CPITN score 4 (severe periodontitis), the AUROCC was 0.72 (95% CI 0.61–0.83; SE 0.06). The optimal predicted probability cut-off value was 0.41. The calibration plot showed that most of the dots were lying close to the reference line, which indicated that there was a good fit between the predicted probability and the actual probability (Fig. 4). The sensitivity of the algorithm in this population at the optimal score was 54% and the specificity was 81%. The PPV was 34% and the NPV scored 81%.

## Discussion

In the current study, two algorithms developed by Verhulst et al. [1] to predict total and severe periodontitis are externally validated in a medical care setting. The CPITN score was used as gold standard. We found that the algorithm for total periodontitis had a sensitivity of 49% and a specificity of 68% when using CPITN score 3 and 4 combined as gold standard to be suspected of having periodontitis. In the study from Verhulst et al. [1], this was 78% and 84%, respectively. The algorithm for severe periodontitis had a sensitivity of 54% and a specificity of 81% when using CPITN score 4 as gold standard to be suspected of having severe periodontitis. In our previous study [1], this was 80% and 70%. Sensitivity and specificity between 60 and 80% are seen a moderate validity [20]. Only the sensitivity of the total periodontitis algorithm was on the low side. A model with a sum of the sensitivity and specificity ≥120 is considered to have a good validity [21]. This is the case for the algorithm for severe periodontitis (sum 135), while for the algorithm for total periodontitis, the sum (117) just failed the threshold of 120. The algorithm for total periodontitis showed an AUROCC of 0.59, which is considered not to be sufficient; the algorithm for severe periodontitis showed an AUROCC of 0.72, which is seen as acceptable [22]. In the study from Verhulst et al. [1], these values were 0.88 and 0.82, respectively, which is seen as excellent [22]. The calibration of the algorithm for severe periodontitis seems to be acceptable based on the calibration plot, which indicated that there is a good agreement between predicted and actual probability. The calibration of the algorithm for total periodontitis seems to be not sufficient based on the calibration plot, which indicated that there is not a good agreement between predicted and actual probability. By combining the outcomes above at this point, the algorithm for severe periodontitis seems to be satisfactory in a medical setting. The algorithm for total periodontitis does not seem to be satisfactory at this moment. The screening in a medical setting for severe periodontitis seems anyway more realistic and essential, as it is severe periodontitis that has major systemic effects such as effects on metabolic control [13].

Although the results from the algorithms in the previous study were good, they perform less good in the external medical care setting. This may be explained by a different gold standard. The previous study used full-mouth probing and clinical attachment loss measurements applying The Centers for Disease Control and Prevention-American Academy of Periodontology (CDC-AAP) for case definition. In the present study, the CPITN was used. Because the overall goal of the algorithm is to screen for people who are “suspected to have (severe) periodontitis,” the CPITN fits well. Performing the CPITN takes just a few minutes which is a time-saving advantage compared with the time for a complete periodontal examination. However, using the CPITN can cause overestimation of the prevalence periodontitis cases, in particular through cases with CPITN score 3. As such, the prevalence of total periodontitis in the current cohort seems somewhat high compared to previously reported prevalences (55% vs 50%, respectively) [2]. Also, the CPITN scoring (in particular CPITN score 3) may have suffered somewhat due to the fact that in the hospital setting no dental equipment was available; on the other hand, we used a good lightning and examined the patient in supine position. Moreover, in the hospital setting, no dental equipment was available. Another reason for the less good performance in the medical setting may be differences in study populations: in Supplementary Table 2 we present characteristics for the current medical study population and the previous dental school population. The populations were statistically different for age, smoking, and diabetes. The mean age in the current study population (55.7 years of age) was higher than in the dental school study population (45.2 years of age) (*p* < 0.001). There were less smokers in the current study population (13.5%) than in the previous study population (23.7%) (*p* = 0.013); there were more diabetes mellitus patients in the current study population (76.3%) compared to the previous study population (23.7%) (*p* < 0.001). Possibly, also the socioeconomic status in the previous and the current study population is different, but this was not tested; socioeconomic status can influence the self-reported oral health [23].

The major strength of this research is the fact that the previously developed algorithms are externally validated. Because the algorithms are made to be implemented in a medical setting, for example, by doctors, general physicians, and nurses, it is important that it performs satisfactory in this specific field. The participants in the hospital had various illnesses such as diabetes mellitus, cardiovascular diseases, HIV, rheumatoid arthritis, hypercholesterolemia, and dyslipidemia. Because the models are made to be used for patients with comorbidities (it is of high importance to screen these people on periodontitis), we have chosen this patient cohort. Thus, at this point, the algorithm for severe periodontitis can be used in an overall medical setting.

PPV and NPV are seen as clinical relevance of a test. For the total periodontitis algorithm, the NPV was 55% and the PPV 57%. For the severe periodontitis algorithm, it was 81% and 34%, respectively. This means that 34% of patients with a positive test result truly had CPITN score 4. In the previous study, a higher PPV (56%) was seen. The PPV can increase with a larger study population including more cases with CPITN score 4. Nonetheless, the algorithm was accurate whether a patient is not suspected to have severe periodontitis. Just a small group (19%) of patients who are “not suspected to have severe periodontitis” do actually have the disease and were not picked out. On the other hand, 66% of the participants who are “predicted to have severe periodontitis,” actually do not have this severity. However, this does not pose a major problem because being predicted to have severe periodontitis leads to advise to visit the dentist who needs to do a final periodontal diagnosis and eventual treatment. Also, the patient may suffer from moderate periodontitis (CPITN score 3) which is good to be noted in a dental visit. Overall, the algorithm for severe periodontitis developed by Verhulst et al. [1] seems to be sufficiently accurate for periodontal screening in a medical setting.

Previous studies [18, 24–28] in the USA, Spain, France, and Germany were performed in a similar way as the study from Verhulst et al. [1]. They all used only SROH questions and demographic data to predict severe periodontitis; no oral examination was needed to predict the periodontal state. The Spanish [25, 26] and French [24] studies used the SROH questions from Eke et al. [18], similar to the current study. The studies from Germany [27, 28] used SROH items developed by the German Society for Periodontology. However, none of these studies, except the one from Zhan et al. [28], performed an external validation. The sensitivity from these prediction models ranged from 75 to 85% and the specificity ranged from 58 to 87%. The different AUROCC values were 0.75–0.86. However, due to differences in case definitions and gold standards, it is difficult to compare these studies. The models which included a combination of SROH questions and demographics performed the best [24–26]. Surprisingly, all the models included another combination of SROH questions and demographic factors. In the former studies, the case definition was based on the CDC-AAP or a periodontal screening tool, while the current study used the CPITN, which may select a different group of “being suspected of having severe periodontitis.”

## Conclusion and recommendation

Because general physicians (especially those who perform diabetes care) are obligated to screen patients for periodontitis, it is our general goal that they can use a prediction model in medical settings without an oral examination. It is time-saving, cost-effective, and does not require any dental knowledge and equipment. The performance of the algorithm for severe periodontitis is found to be sufficient in the current medical study population. Further external validation of periodontitis algorithms in a medical setting is planned and will follow soon to investigate how the current algorithms may need recalibration and adjustments.

### Supplementary Information

ESM 1 (DOCX 20 kb)
