# Correction to: External validation of a rapid, non-invasive tool for periodontitis screening in a medical care setting

**DOI:** 10.1007/s00784-023-05318-2

**Published:** 2023-11-06

**Authors:** N. Nijland, F. Overtoom, V. E. A. Gerdes, M. J. L. Verhulst, N. Su, B. G. Loos

**Affiliations:** 1 Present Address: Department of Periodontology, Academic Center for Dentistry Amsterdam (ACTA), University of Amsterdam and Vrije Universiteit Amsterdam, Gustav Mahlerlaan 3004, 1081 LA Amsterdam, The Netherlands; 2 Department of Vascular Medicine, Amsterdam University Medical Center, Amsterdam, The Netherlands; 3 Sunstar Suisse, Etoy, Switzerland; 4 Department of Oral Public Health, Academic Center for Dentistry Amsterdam (ACTA), University of Amsterdam and Vrije Universiteit, Amsterdam, The Netherlands

**Correction to: Clinical Oral Investigations**

10.1007/s00784-021-03952-2

In the published paper, the authors discovered an error in the number of patients belonging to the category CPITN 4 (severe periodontitis). The number of patients with CPITN 4 was reported to be 38; however, the correct number should be 24. This mistake occurred due to the conversion from the Dutch Periodontal Screening Index (DPSI) to CPITN. In the medical setting, they screened using the DPSI. The DPSI, used in the Netherlands, is a modification of the CPITN for score 3; this screening method differentiates between patients with pockets 4–5 mm without gingival recessions (DPSI 3-) and pockets 4–5 mm with gingival recession (DPSI 3 +) [2]. In the Dutch system, patients with DPSI 3 + and 4 are required to have extensive diagnostic procedures because they may suffer from severe periodontitis [2]. Due to this reason, they have incorrectly grouped DPSI 3 + and 4 into CPITN 4, while patients with a DPSI 3 + score (*n* = 14) should belong to the CPITN 3 group. Thus, the results, which are relevant to severe periodontitis, need to be reported correctly. The corrected results (including corresponding tables and figures) of the original paper will also be presented. Importantly, the discussion, conclusions, and implications of the study have not changed, and the meaning of the original paper remains as before. The authors would like to apologize for any inconvenience caused.

The original article has been corrected.

