# Supplementary material for: External validation of a rapid, non-invasive tool for periodontitis screening in a medical care setting
Source: Clin Oral Investig. 2021 May 12;25(12):6661–9. doi: 10.1007/s00784-021-03952-2 (PMC8602137; doi:10.1007/s00784-021-03952-2)
Supplement: Supplementary file 1 — (DOCX 20 kb) [file 784_2021_3952_MOESM1_ESM.docx]

**Supplementary table 1**. Self-reported oral health (SROH) questionnaire with Dutch translation, abbreviations and answer possibilities.

| Abbreviation | Question with Dutch translation | Answers |
| --- | --- | --- |
| Q1. Gum disease | Do you think you might have gum disease?  *Denkt u dat u een tandvleesaandoening (gingivitis of parodontitis) heeft?* | Yes, no |
| Q2. Own teeth/ gum health | Overall, how would you rate the health of your teeth and gums?  *Hoe zou u over het algemeen genomen de gezondheid van uw tanden en tandvlees beoordelen?* | Poor, fair, good, very good, excellent |
| Q3. Gum treatment | Have you ever had treatment for gum disease such as scaling and root planing, sometimes called “deep cleaning”?  *Bent u wel eens voor een tandvleesaandoening behandeld, soms ook wel “diep schoonmaken” of “pocket behandeling” genoemd?* | Yes, no |
| Q4. Loose teeth | Have you ever had any teeth become loose on their own, without an injury?  *Heeft u wel eens last van losstaande tanden gehad, zonder dat daar een ongeluk of trauma aan vooraf ging?* | Yes, no |
| Q5. Bone loss | Have you ever been told by a dental professional that you lost bone around your teeth?  *Heeft een tandheelkundige zorgverlener (tandarts, mondhygiënist, enz.) u wel eens verteld dat u botverlies heeft rondom uw tanden?* | Yes, no |
| Q6. Tooth appearance | During the past three months, have you noticed a tooth that doesn’t look right?  *Heeft u de afgelopen drie maanden wel eens gemerkt dat een tand er niet goed uit zag?* | Yes, no |
| Q7. Floss use | Aside from brushing your teeth with a toothbrush, in the last seven days, how many times did you use dental floss or any other device to clean between your teeth?  *Hoe vaak heft u de afgelopen zeven dagen, afgezien van tandenpoetsen met een normale tandenborstel, geflost of op een andere manier tussen uw tanden schoongemaakt?* | ….. days per week |
| Q8. Mouthwash use | Aside from brushing your teeth with a toothbrush, in the last seven days, how many times did you use mouthwash or other dental rinse product that you use to treat dental disease or dental problems?  *Hoe vaak heft u de laatste zeven dagen, afgezien van tandenpoetsen met een normale tandenborstel, mondspoelmiddel of een ander spoelproduct gebruikt om tandheelkundige ziekte of klachten te behandelen?* | …. days per week |

**Supplementary table 2**: Comparison of the previous and current study population

|  | **Total population** | **Previous study population**^a^ | **Current study population** | **P-value**^c^ |
| --- | --- | --- | --- | --- |
| N (%) | 311 (100) | 156 (50.2) | 155 (49.8) |  |
| Age (years) | 50.5 | 45.2 ± 16.4^b^ | 55.7 ± 15.6 | **<0.001^*^** |
| Sex |  |  |  |  |
| Male | 171 (55) | 86 (55.1) | 85 (54.8) | 0.959 |
| Female | 140 (45) | 70 (44.9) | 70 (45.2) |  |
| Smoking (current) | 58 (18.6) | 37 (23.7) | 21 (13.5) | **0.013^*^** |
| Diabetes mellitus | 38 (12.2) | 9 (5.8) | 29 (18.7) | **<0.001^*^** |

*Data are presented as either mean ± SD or n (%)*

*^a^Study population from Verhulst et al. [1] taken in a dental school*

^b^*Data of six patients were missing and not taken into analysis*

^c^*P-values were obtained from independent-samples t-test (continuous data) or Chi-square test (categorial data)*

*^*^Statistically significant with p <0.05*
